# Do personal resilience, coping styles, and social support prevent future psychological distress when experiencing workplace bullying? Evidence from a 1-year prospective study

**DOI:** 10.1186/s40359-022-00991-6

**Published:** 2022-12-16

**Authors:** Kanami Tsuno

**Affiliations:** 1 School of Health Innovation, Kanagawa University of Human Services, Research Gate Building TONOMACHI2, 3-25-10 Tonomachi, Kawasaki-ku, Kawasaki, Kanagawa 210-0821 Japan; 2 Department of Mental Health, Graduate School of Medicine, The University of Tokyo, Tokyo, Japan

**Keywords:** Buffering effect, Organizational support, Mental health, Workplace bullying, Worker

## Abstract

**Background:**

Although previous studies have identified that workplace bullying causes serious mental health problems to the victims, it is not yet fully investigated moderating factors on the association between workplace bullying and psychological distress. This longitudinal study, therefore, examined the moderating role of organizational resources such as supervisor support or coworker support as well as individual resources such as stress coping styles or personal resilience on the association.

**Methods:**

A prospective cohort study for 2036 civil servants was conducted with a one-year time lag (follow-up rate: 77.2%). At baseline, Negative Acts Questionnaire-Revised, Connor–Davidson Resilience Scale, Brief Scales for Coping Profile, and Brief Job Stress Questionnaire were used to measure workplace bullying, personal resilience, stress coping styles, and social support, respectively. Psychological distress was measured using K6 both at baseline and follow-up.

**Results:**

The results of hierarchical multiple regression analyses showed that workplace bullying was associated with subsequent increased psychological distress even after adjusting for individual and occupational characteristics, but its association disappeared after adjusting for psychological distress at baseline. After adjusting for psychological distress at baseline, greater resilience, greater seeking help, greater changing view, and lower avoidance were associated with lower subsequent psychological distress when being bullied. In contrast, worksite social support and family/friends support was not associated with lower subsequent psychological distress when being bullied. A significant interaction effect of workplace bullying and changing mood was observed on subsequent psychological distress.

**Conclusions:**

The effects or moderating factors were limited on the longitudinal association between bullying and mental health because psychological distress at baseline was the strongest predictor of subsequent psychological distress.

## Background

Workplace bullying, defined as “repeated actions and practices that are directed against one or more workers, that are unwanted by the target, that may be carried out deliberately or unconsciously, but clearly cause humiliation, offense and distress, and that may interfere with work performance and/or cause an unpleasant working environment” [1, 2], is one of the significant job stressors in the work environments. According to a meta-analysis [3], about 15% of employees have experienced bullying. Empirical studies in Japan showed that about 9.0–15.5% of employees have experienced bullying during the past six months [4–6], which is similar to European countries (9.5% in Scandinavia and 15.7% in other European countries) [3].

Various health outcomes have been reported as the consequences of workplace bullying [7]. For example, a cross-sectional study of 2194 Japanese civil servants showed that victims who experienced bullying on a weekly basis had 8 times higher risk of psychological distress and 12 times higher risk of post-traumatic stress symptoms compared to non-victims, after adjusting for age, sex, education, chronic disease, occupation, employment status, shift work, overtime working hours during the past month [8]. A two-year prospective cohort study of 543 workers at welfare facilities for the elderly showed that person-related bullying was significantly linked to subsequent psychological and physical stress responses [9].

Although previous studies have already identified that workplace bullying causes serious mental health problems to the victims, it is not yet fully investigated whether there are moderating factors on the association. To prevent adverse mental health outcomes, identifying moderators is needed. Therefore, this study investigates the moderating effects of individual resources such as personal resilience and coping styles and organizational resources such as worksite social support on the association between workplace bullying and psychological distress in a longitudinal design.

### Organizational and individual resources which moderate the association between workplace bullying and mental health

#### Worksite social support

As one of the organizational resources, worksite social support is a key component of the demand-control-support model, which defined a combination of high job demands, low job control, and low worksite social support as the most stressful situation [10, 11]. Worksite social support usually consists of supervisor support and coworker support and a contributor of buffering psychological distress among workers under stressful working environments such as job strain (a combination of high job demands and low job control). Worksite social support is defined as the degree to which individuals receive supports from supervisors and coworkers. Receiving a good amount of supports from work members prevent workers from developing mental health issues. Besides that, supervisor and coworker support can increase the employee’s comfort in the organization by satisfying needs such as self-esteem, acceptance, and belonging [12].

Previous research already confirmed a negative relationship between workplace bullying and both supervisor and coworker support [4, 13, 14]. On the other hand, inconsistent results were found in the moderating effects on the relationship between workplace bullying and mental health outcomes. For example, a cross-sectional study of 820 employees from various organizations in Poland has reported that only coworker support moderates the relationship [14], while another cross-sectional study of 1733 employees in New Zealand reported that both supervisor and coworker support reduced psychological strain among workers who experienced workplace bullying [15]. Although a cross-sectional study of 335 school teachers in Australia reported perceived organizational support moderated the relationship between bullying and intention to leave [16], a cross-sectional study of 222 employees from various organizations in the UK found a weaker moderating effect of support from senior management [13]. Additionally, even coworker support is not enough to reduce distress when individuals experienced high levels of workplace bullying because workplace bullying itself has a strong effect on mental health beyond worksite social support [13, 14]. It remains unclear whether specific forms of social support (e.g., supervisor or co-worker support) have a greater protective effect against workplace bullying. Also, there is no study that investigated these associations in a longitudinal design.

##### H1

Social support from supervisors, co-workers, and family/friends moderates the relationship between employees’ exposure to workplace bullying and their subsequent psychological distress.

#### Personal resilience

Although the definition of resilience varies in the literature, resilience, in general, refers to one’s ability to protect oneself from stressful life events, especially life-changing adverse events. For example, Connor and Davidson explained that “resilience embodies the personal qualities that enable one to thrive in the face of adversity” [17]. On the other hand, Fletcher and Sarkar defined psychological resilience as “the role of mental processes and behavior in promoting personal assets and protecting an individual from the potential negative effect of stressors” [18]. Resilient people tend to have high self-efficacy, past successes, a sense of control, a sense of humor, patience, optimism, supports from others, or personal goals in their lives [17]. According to Wagnild [19]’s model, a person is resilient when he or she has a personal purpose in life, when he or she is determined to combat adversity to achieve his or her goal, when he or she keeps a balanced perspective on life, and when he or she uses humor to deal with life's stressors. Resilience enables one to thrive in the face of adversity such as combats or natural disasters and prevents one from developing mental illness such as psychological distress or post-traumatic stress disorder [17, 20]. Although resilience is often considered as personal characteristics or traits, it has been reported as one of the two mental capital which individuals can obtain in their life course [21]. A systematic review of work-based interventions also indicates training such as Cognitive Behavior Therapy, self-regulation techniques, coaching sessions, etc. can improve personal resilience [22].

Experiencing workplace bullying is one of the adverse life events in working lives. Several studies have found a negative correlation between personal resilience and workplace bullying, as well as negative correlations between resilience and mental health outcomes [23]. Recent cross-sectional studies have shown that resilience plays a mediating or moderating role in the relationship between workplace bullying and employees’ psychological health, emotional exhaustion, or physical strain [24–26]. These results showed that those employees who have higher levels of resilience have lower levels of emotional exhaustion, distress, or physical strain when they were exposed to workplace bullying. However, whether personal resilience prevents subsequent psychological distress among workers who experienced workplace bullying is unexplored in a longitudinal study.

##### H2

Personal resilience moderates the relationship between employees’ exposure to workplace bullying and their subsequent psychological distress.

#### Stress coping styles

Coping skills are the other mental capital that moderate stress reactions and individuals can obtain in their life course, in addition to personal resilience [21]. Original formulations of coping strategies consist of two dimensions: problem-focused coping (taking action and information seeking) and emotion-focused coping (change the way in which one thinks or feels a stressful situation, avoidance, and denial) [27]. Seeking help and taking action are conceptualized as active strategies that may reduce stress reactions when individuals experienced stressful events, whereas avoidance and doing nothing are passive strategies that may increase stress reactions. In contrast to situation-specific coping strategies, coping styles refer to stable dispositional characteristics that reflect generalized tendencies to interpret and respond to stress [28]. As reported that resilience may also be viewed as a measure of successful stress coping ability [17], individual differences in coping styles may act as moderators of the impact of stress on the results of stressful events.

Victims of workplace bullying are less likely to use problem-solving and more likely to use avoidance or resignation strategies than non-victims [29]. The study in Iceland suggests that active coping styles are used at the beginning of bullying, but victims use more passive coping strategies as bullying becomes more serious [30]. Previous research suggests different coping strategies have different effects on the association between workplace bullying and mental health. For instance, Bernstein and Trimm [31] reported that seeking help and assertiveness moderated the relationship between bullying and psychological well-being, while avoidance and doing nothing negatively impact its relationship. A systematic review [32] has reported that reappraisal coping, confrontive coping, practical coping, direct coping, active coping, social support, and self-care had a buffer-effect on the association between work stressors and bullying, while wishful thinking, emotional coping, avoidance, recreation, social support, and suppression had a boost effect on this association. However, a causal relationship remains unclear due to the cross-sectional design in the literature. Whether positive coping styles prevent employees from developing subsequent psychological distress after they experienced workplace bullying has not been investigated.

##### H3

Positive coping styles moderates the relationship between employees’ exposure to workplace bullying and their subsequent psychological distress.

## Methods

### Participants and procedure

A prospective cohort study was conducted for all employees in public sectors in one city located in the Greater Tokyo Area in Japan (N = 3142). This study was conducted as a part of the Working Conditions and Stress Survey. The questionnaires were distributed through the human resource department with a letter describing the aims and procedure of the study assuring that the survey was voluntary and no individual would be identified in reporting the data. Each questionnaire was returned in a sealed envelope, collected at the human resource department, and then sent to the University of Tokyo. The author opened the envelopes, stored the questionnaires in a locked room after data entry, and shredded and discarded them after completing the research. Employee ID was collected to combine the baseline and follow-up data but deleted after the combination completed. Thus, the data were analyzed anonymously. The details of the study procedures have been reported elsewhere [33].

At baseline (T1), a total of 2638 participants answered the questionnaire (response rate: 83.9%). One-year later (T2), 2036 participants completed follow-up survey (follow-up rate: 77.2%). The reason for drop-out is unknown because the survey was non-mandatory and we did not ask why they did not participate in the follow-up survey.

## Measurements

### Workplace bullying

Workplace bullying was measured using the 22-item Negative Acts Questionnaire-Revised (NAQ-R) [4, 34]. NAQ-R assesses how often respondents have experienced a variety of bullying behaviors in the previous six months. Items were scored on a 5-point Likert scale ranging from 1 = never to 5 = daily. The item examples are “someone withholding information which affects your performance” and “persistent criticism of your work and effort.” In the present study, a NAQ-R sum-scale was used in the statistical analyses; a higher score means frequent workplace bullying. Cronbach’s alpha was 0.92 in this study, which shows high internal consistency.

### Psychological distress

Psychological distress was measured using the K6 [35, 36], which consists of six items asking how often respondents have experienced symptoms of psychological distress during the last 30 days. The item examples are “hopeless” and “so depressed that nothing could cheer you up.” Items were scored on a 5-point Likert scale ranging from 0 = never to 4 = daily and a K6 sum scale was used for statistical analyses; a higher score means having greater psychological distress. Cronbach’s alpha was 0.90 at baseline and 0.91 at follow-up in this study.

### Organizational resource

#### Worksite social support

Worksite social support was assessed by the sub-scale of the Brief Job Stress Questionnaire (BJSQ) [37]. BJSQ consists of 57 items that measure various types of job stressors, psychological and physical symptoms, and social support. Among them, the author used two sets of three items for supervisor support and co-worker support. Items are “how freely can you talk with your supervisors/co-workers?” “how reliable are your supervisors/co-workers when you are troubled?” or “how reliable are your supervisors/co-workers when you are troubled?” Items were scored on a 4-point Likert scale ranging from 1 = rarely to 4 = almost always and each sum scale was used for statistical analyses; a higher score means receiving greater support from supervisors or co-workers. Cronbach’s alpha was 0.81 for supervisor support and 0.80 for coworker support in this study.

### Individual resources

#### Family and friends support

Family and friends’ support was also assessed by the sub-scale of the Brief Job Stress Questionnaire (BJSQ) [37]. Both support was assessed by three items (“how freely can you talk with your family, friends, etc.?” “how reliable are your family, friends, etc. when you are troubled?” or “how reliable are your family, friends, etc. when you are troubled?”). Items were scored on a 4-point Likert scale ranging from 1 = rarely to 4 = almost always and each sum scale was used for statistical analyses; a higher score means receiving greater support from family/friends. Cronbach’s alpha was 0.79 in this study.

#### Personal resilience

Personal resilience was assessed with the Connor-Davidson Resilience Scale (CD-RISC), which consists of 25 items asking how the person has felt over the past month [17]. Item examples are “able to adapt to change” and “tend to bounce back after illness or hardship.” Items were scored on a 5-point Likert scale ranging from 0 = not true at all to 4 = true nearly all the time and a CD-RISC sum scale was used for statistical analyses; a higher score means having greater personal resilience. In this study, Cronbach’s alpha was 0.94.

#### Coping styles

Coping Styles were assessed with the Brief Scales for Coping Profile (BSCP) [38], which was specifically developed for measuring non-situation-specific coping styles among workers. BSCP consists of 18 items describing six coping styles; active solution (“I try to analyze the causes and solve the problem.”), changing mood (“I try to do something that calms me down.”), seeking help for a solution (I consult someone who is very familiar with the problem.), emotional expression involving others (“I complain to people who have nothing to do with the problem.”), avoidance (“I let time go, thinking passively that the situation will change someday.”), and changing a point of view (“I try to think this experience is good for me.”). Among these, active solution and seeking help has been regarded as positive coping styles [38]. Others are emotion-based coping styles, which are positively correlated with mental health outcomes. Each coping style consists of three items. Items were scored on a 4-point Likert scale ranging from 1 = never to 4 = often and a sum-scale of each subcategory was used for statistical analyses. Reliability and validity of the scale have been reported and shown a good level of reliability and validity [38]. In this study, Cronbach’s alpha was 0.86 for active solution, 0.84 for seeking help, 0.85 for changing mood, 0.45 for emotional expression, 0.71 for avoidance, and 0.76 for changing view.

### Other covariates

Participants answered questions at baseline regarding individual characteristics such as gender, educational status, marital status, and chronic condition (whether receiving medical treatment for chronic disease(s)), as well as occupational characteristics such as occupation and employment status. Life events during the previous year were accessed at follow-up, including job promotion, marriage, divorce, injury, or family member’s disease or death. Participants who responded “Yes” to at least one life event were classified as having had life events during follow-up.

### Statistical analysis

Hierarchical multiple regression analyses were conducted to examine the effect of organizational resources such as worksite social support and individual resources such as family and friends support, personal resilience, and coping styles on psychological distress. First, exposure to workplace bullying and various participants’ individual and occupational characteristics at T1 including life events during the follow-up were entered the model (Model 1). Second, all the occupational and individual resources at T1 were additionally entered the model to examine the main effects of these variables (Model 2). Third, psychological distress at baseline was additionally entered the model (Model 3). Finally, all interaction variables (bullying x each occupational and individual resource) were simultaneously entered the model (Model 4). The same procedure was applied for coping styles. The level of significance used was 0.05 (two-tailed). SPSS 27.0 J for Windows (IBM, Japan) was used for the statistical analyses.

## Results

### Participants characteristics

Table 1 provides basic characteristics for the participants in this study (N = 2036). Overall, most of the participants were college graduates, currently married, administrators/clerks, and full-time non-managerial or non-shift employees. Dropouts were more likely to be females, junior college/technical school graduates, currently married, having chronic diseases, nurses, and shift workers. Moreover, dropouts were older than follow-ups and re-employed after retirement, indicting some baseline participants retired during the follow-up.

**Table 1:** Baseline sample characteristics (*N* = 2638)

Variable	Follow-up				Drop-out				*p* value
	*n*	%	Mean	SD	*n*	%	Mean	SD	
*Demographic characteristics*									
Gender									< 0.01
Male	1142	57.6			230	39.4			
Female	894	42.4			354	60.6			
Age (years)			42.8	11.4			46.1	12.5	< 0.01
20–24	124	6.1			23	4.0			
25–29	205	10.1			45	7.9			
30–34	215	10.6			60	10.5			
35–39	298	14.6			71	12.4			
40–44	250	12.3			69	12.1			
45–49	222	10.9			42	7.4			
50–54	333	16.3			65	11.4			
55–59	290	14.2			88	15.4			
Over 60	99	4.9			108	18.9			
Educational status									< 0.01
Under high school graduate	563	27.7			157	26.9			
Junior college/technical school graduate	671	33.0			228	39.0			
University/graduate school graduate	802	39.4			199	34.1			
Marital status									0.010
Currently married	1498	73.6			448	79.0			
Never married/divorced/widowed	538	26.4			119	21.0			
Having chronic disease									0.322
Yes	445	21.9			138	23.6			
No	1591	78.1			446	76.4			
*Occupational characteristics*									
Occupation									< 0.01
Administrator/clerk	741	36.4			168	29.3			
Technician	189	9.3			23	4.0			
Fieldworker^‡^	268	13.2			88	15.3			
Nursery staff	262	12.9			83	14.5			
Public health nurse/nutritionist	153	7.5			74	12.9			
Medical technician	54	2.7			17	3.0			
Hospital nurse/midwife	83	4.1			71	12.4			
Fire defense personnel	240	11.8			27	4.7			
Others	44	2.2			23	4.0			
Employment status									< 0.01
Manager	41	2.0			18	3.1			
Middle manager	179	8.9			31	5.3			
Assistant manager	571	58.5			118	20.2			
General employee	942	47.0			228	39.0			
Re-employment after retirement	53	2.6			70	12.0			
Part-time	194	9.7			105	18.0			
Others	24	1.2			14	2.4			
Shift work									0.014
Yes	605	29.7			176	35.3			
No	1431	70.3			322	64.7			
*Life event(s) during follow-up*									
Yes	1249	61.3							
No	787	38.7							
*Scale scores at T1*									
Workplace bullying			25.9	7.4			27.0	9.8	< 0.01
Supervisor support			8.8	2.3			8.4	2.3	< 0.01
Co-worker support			9.6	2.0			9.3	2.0	< 0.01
Family/friends support			8.8	2.3			8.9	2.3	0.276
Active solution			9.2	2.3			8.7	2.5	< 0.01
Seeking help			8.2	2.5			7.8	2.6	< 0.01
Changing mood			7.9	2.6			7.6	2.7	0.023
Emotional expression			5.1	1.6			5.1	1.6	0.401
Avoidance			5.3	1.9			5.4	2.1	0.383
Changing view			7.4	2.2			7.3	2.5	0.502
Resilience			51.5	14.7			50.1	15.8	0.055
Psychological distress			5.8	5.1			6.6	5.7	< 0.01

*SD* Standard deviation

^‡^Field worker includes sanitation worker, school food service worker, school janitor, telephone exchange operator, etc.

### Correlation between variables

Table 2 shows Pearson’s correlation coefficients between workplace bullying, individual and occupational resources, and psychological distress. Workplace bullying at T1 was associated with all variables except for active solution and changing view. Psychological distress at T1 and T2 were associated each other (r = 0.60).

**Table 2:** Pearson’s correlation coefficients between the total score of NAQ-R, individual and occupational resources, and psychological distress

	Variables	Range	Mean (SD)	Alpha	1	2	3	4	5	6	7	8	9	10	11	12
1	Workplace bullying at T1	22–88	26.0 (7.4)	0.92	1.00											
2	Supervisor support at T1	3–12	8.7 (2.3)	0.81	− 0.30**	1.00										
3	Co-worker support at T1	3–12	9.6 (2.0)	0.80	− 0.27**	0.53**	1.00									
4	Family/friends support at T1	3–12	8.8 (2.3)	0.79	− 0.06**	0.27**	0.34**	1.00								
5	Active solution at T1	3–12	9.2 (2.3)	0.86	0.01	0.20**	0.22**	0.16**	1.00							
6	Seeking help at T1	3–12	8.2 (2.5)	0.84	− 0.08**	0.33**	0.34**	0.24**	0.58**	1.00						
7	Changing mood at T1	3–12	7.9 (2.6)	0.85	0.07**	0.10**	0.15**	0.24**	0.26**	0.25**	1.00					
8	Emotional expression at T1	3–12	5.1 (1.6)	0.45	0.17**	− 0.01	0.08**	0.18**	0.13**	0.27**	0.33**	1.00				
9	Avoidance at T1	3–12	5.3 (1.9)	0.71	0.27*	− 0.11**	− 0.10**	− 0.06**	− 0.02	− 0.08**	0.15**	0.30**	1.00			
10	Changing view at T1	3–12	7.4 (2.2)	0.76	− 0.04	0.20**	0.23**	0.19**	0.36**	0.34**	0.37**	0.17**	0.16	1.00		
11	Resilience at T1	0–96	51.5 (14.7)	0.94	− 0.11**	0.28**	0.33**	0.33**	0.38**	0.32**	0.27**	0.06*	− 0.22**	0.47**	1.00	
12	Psychological distress at T1	0–24	5.8 (5.1)	0.90	0.45**	− 0.26**	− 0.27**	− 0.13**	− 0.05*	− 0.11**	− 0.01	0.17**	0.34	− 0.14**	− 0.36**	1.00
13	Psychological distress at T2	0–24	5.2 (5.1)	0.91	0.27**	− 0.14**	− 0.19**	− 0.11**	− 0.03	− 0.08**	− 0.01	0.14**	0.28**	− 0.12**	− 0.29**	0.60**

*p < 0.05; **p < 0.001

### Main and interaction effects of workplace bullying and organizational/individual resources on psychological distress

Table 3 presents the results from the hierarchical multiple regression analyses. Model 1 consists of workplace bullying and individual and occupational characteristics. In this model, workplace bullying was linked to subsequent greater psychological distress (*β* = 0.24, *p* < 0.01). In Model 2 where individual and organizational resources at T1 were additionally entered, workplace bullying remained significant (*β* = 0.19, *p* < 0.01) and greater co-worker support and greater resilience were associated with subsequent lower psychological distress (*β* =  − 0.05, *p* < 0.05; − 0.23, *p* < 0.01, respectively). Of the individual factors, women, young workers, managers, and those who experienced at least one life event during follow-up were more likely to experience psychological distress at a one-year follow-up. In Model 3 where psychological distress at T1 was additionally entered, the main effect of workplace bullying on subsequent psychological distress disappeared and psychological distress at T1 was the strongest predictor for subsequent psychological distress. In Model 4 where interaction variables were additionally added, no significant interaction was found to be with subsequent psychological distress.

**Table 3:** Buffering effects of individual and occupational resources on the association between workplace bullying and subsequent psychological distress: hierarchical multiple regression

	Model 1			Model 2			Model 3			Model 4		
	b	SE	*β*	b	SE	*β*	b	SE	*β*	b	SE	*β*
*Step 1*												
Exposure to workplace bullying at T1	0.16	0.02	0.24**	0.13	0.02	0.19**	0.01	0.02	0.01	0.03	0.02	0.05
Gender (male = 1, female = 0)	− 1.37	0.31	− 0.14**	− 1.39	0.30	− 0.14**	− 0.86	0.27	− 0.09**	− 0.88	0.27	− 0.09**
Age (years)	− 0.04	0.01	− 0.09**	− 0.05	0.01	− 0.10**	− 0.04	0.01	− 0.08**	− 0.04	0.01	− 0.08**
College degree (yes = 1, no = 0)	0.04	0.27	0.00	0.19	0.26	0.02	0.07	0.23	0.01	0.06	0.23	0.01
Marital status (married = 1, unmarried = 0)	− 0.65	0.28	− 0.06*	− 0.47	0.27	− 0.04	− 0.26	0.24	− 0.02	− 0.24	0.24	− 0.02
Having chronic disease (yes = 1, no = 0)	0.47	0.29	0.04	0.25	0.28	0.02	− 0.06	0.25	− 0.01	− 0.07	0.25	− 0.01
Administrator/clerk (yes = 1, no = 0)	1.15	0.80	0.11	0.91	0.77	0.09	0.96	0.69	0.09	0.95	0.69	0.09
Technician (yes = 1, no = 0)	0.84	0.88	0.05	0.46	0.84	0.03	0.63	0.75	0.04	0.61	0.75	0.04
Fieldworker (yes = 1, no = 0)	0.07	0.85	0.00	− 0.23	0.82	− 0.02	0.27	0.73	0.02	0.26	0.73	0.02
Nursery staff (yes = 1, no = 0)	− 0.74	0.84	− 0.05	− 0.47	0.81	− 0.03	− 0.18	0.72	− 0.01	− 0.16	0.72	− 0.01
Public health nurse/nutritionist (yes = 1, no = 0)	1.34	0.90	0.07**	0.85	0.87	0.04	0.59	0.77	0.03	0.53	0.77	0.03
Medical technician (yes = 1, no = 0)	0.77	1.03	0.03	− 0.05	0.99	0.00	0.26	0.88	0.01	0.29	0.88	0.01
Hospital nurse/midwife (yes = 1, no = 0)	2.61	0.99	0.10	1.99	0.96	0.08*	1.03	0.85	0.04	0.97	0.85	0.04
Fire defense personnel (yes = 1, no = 0)	0.44	0.90	0.03	0.22	0.86	0.01	0.69	0.77	0.05	0.68	0.77	0.05
Manager (yes = 1, no = 0)	0.66	0.32	0.06*	0.62	0.31	0.06*	0.33	0.28	0.03	0.33	0.28	0.03
Shift work (yes = 1, no = 0)	0.28	0.33	0.03	0.37	0.32	0.03	0.17	0.28	0.02	0.14	0.28	0.01
Life event(s) during follow-up (yes = 1, no = 0)	1.42	0.23	0.14**	1.45	0.22	0.14**	0.83	0.20	0.08**	0.85	0.20	0.08**
*Step 2*												
Supervisor support at T1				− 0.05	0.06	− 0.02	0.01	0.05	0.00	0.03	0.05	0.01
Co-worker support at T1				− 0.13	0.07	− 0.05*	− 0.06	0.06	− 0.03	− 0.08	0.06	− 0.03
Family/friends support at T1				− 0.05	0.06	− 0.02	− 0.04	0.05	− 0.016	− 0.04	0.05	− 0.02
Resilience at T1				− 0.08	0.01	− 0.23**	− 0.03	0.01	− 0.09**	− 0.03	0.01	− 0.09**
*Step 3*												
Psychological distress at T1							0.51	0.02	0.51**	0.50	0.02	0.51**
*Step 4*												
Bullying × supervisor support										0.06	0.11	0.01
Bullying × co-worker support										0.17	0.10	0.04
Bullying × family/friends support										− 0.24	0.15	− 0.04
Bullying × resilience										0.10	0.11	0.02
R^2^			0.144**			0.201**			0.370**			0.372**
ΔR^2^			0.144			0.066			0.168			0.004
F change			17.06**			36.20**			464.76**			2.10

b, Partial regression coefficient; *β*, Standard partial regression coefficient; R^2^, Coefficient of determination

*p < 0.05; **p < 0.01

### Main and interaction effects of workplace bullying and coping styles on psychological distress

Similar results were obtained in terms of coping styles (Table 4). Model 1 consists of workplace bullying, individual and occupational characteristics, and coping styles. In this model, seeking help, emotional expression, avoidance, and changing views were associated with subsequent psychological distress. In Model 2 where psychological distress at T1 was additionally entered, the main effect of workplace bullying on subsequent psychological distress disappeared. In Model 3 where interaction variables additionally entered, seeking help, avoidance, and changing view remained significant with increased psychological distress. However, only one significant interaction (bullying x changing mood) was found to be associated with subsequent psychological distress (*β* = 0.05, *p* < 0.05).

**Table 4:** Buffering effects of coping styles on the association between workplace bullying and psychological distress: hierarchical multiple regression

	Model 1			Model 2			Model 3		
	b	SE	*β*	b	SE	*β*	b	SE	*β*
*Step 1*									
Exposure to workplace bullying at T1	0.12	0.02	0.17**	0.00	0.02	0.00	0.01	0.02	0.02
Gender (male = 1, female = 0)	− 1.12	0.31	− 0.11**	− 0.74	0.28	− 0.07**	− 0.72	0.28	− 0.07**
Age (years)	− 0.05	0.01	− 0.12**	− 0.04	0.01	− 0.09**	− 0.04	0.01	− 0.09**
College degree (yes = 1, no = 0)	0.08	0.27	0.01	0.02	0.23	0.00	0.03	0.24	0.00
Marital status (married = 1, no = 0)	− 0.38	0.28	− 0.03	− 0.22	0.24	− 0.02	− 0.24	0.24	− 0.02
Having chronic disease (yes = 1, no = 0)	0.42	0.28	0.03	− 0.01	0.25	0.00	0.02	0.25	0.00
Administrator/clerk (yes = 1, no = 0)	0.88	0.78	0.09	0.94	0.69	0.09	0.96	0.69	0.09
Technician (yes = 1, no = 0)	0.57	0.85	0.03	0.66	0.75	0.04	0.66	0.75	0.04
Fieldworker (yes = 1, no = 0)	− 0.21	0.83	− 0.01	0.27	0.73	0.02	0.32	0.74	0.02
Nursery staff (yes = 1, no = 0)	− 0.50	0.82	− 0.03	− 0.20	0.72	− 0.01	− 0.15	0.73	− 0.01
Public health nurse/nutritionist (yes = 1, no = 0)	0.96	0.88	0.05	0.60	0.77	0.03	0.61	0.78	0.03
Medical technician (yes = 1, no = 0)	− 0.12	1.00	0.00	0.18	0.89	0.01	0.21	0.89	0.01
Hospital nurse/midwife (yes = 1, no = 0)	2.14	0.97	0.08*	1.07	0.86	0.04	1.16	0.86	0.04
Fire defense personnel (yes = 1, no = 0)	− 0.01	0.87	0.00	0.56	0.77	0.04	0.57	0.78	0.04
Manager (yes = 1, no = 0)	0.81	0.32	0.08**	0.42	0.28	0.04	0.45	0.28	0.04
Shift work (yes = 1, no = 0)	0.36	0.32	0.03	0.18	0.28	0.02	0.16	0.28	0.02
Life event(s) during follow-up (yes = 1, no = 0)	1.41	0.23	0.14**	0.80	0.20	0.08**	0.78	0.20	0.08**
Active solution at T1	0.09	0.06	0.04	0.07	0.05	0.03	0.08	0.05	0.04
Seeking help at T1	− 0.19	0.06	− 0.09**	− 0.12	0.05	− 0.06*	− 0.13	0.05	− 0.06*
Changing mood at T1	− 0.08	0.05	− 0.04	− 0.04	0.04	− 0.02	− 0.04	0.04	− 0.02
Emotional suppression at T1	0.17	0.08	0.05*	0.08	0.07	0.02	0.08	0.07	0.03
Avoidance suppression at T1	0.48	0.06	0.18**	0.19	0.06	0.07**	0.19	0.06	0.07**
Changing view at T1	− 0.275	0.057	− 0.12**	− 0.10	0.05	− 0.05*	− 0.10	0.05	− 0.05*
*Step 2*									
Psychological distress at T1				0.51	0.02	0.52**	0.51	0.02	0.51**
*Step 3*									
Bullying × active solution							− 0.18	0.13	− 0.03
Bullying × seeking help							0.13	0.12	0.03
Bullying × changing mood							0.24	0.11	0.05*
Bullying × emotional expression							− 0.16	0.11	− 0.03
Bullying × avoidance							− 0.09	0.11	− 0.02
Bullying × changing view							− 0.03	0.11	− 0.01
R^2^			0.198**			0.377**			0.380**
ΔR^2^			0.055			0.180			0.003
F change			19.52**			493.31**			1.41

b, Partial regression coefficient; *β*, Standard partial regression coefficient; R^2^, Coefficient of determination

*p < 0.05; **p < 0.01

## Discussion

The current prospective cohort study aimed to investigate the moderating effects of individual resources such as personal resilience and coping styles as well as organizational resources such as worksite social support on the association between workplace bullying and subsequent psychological distress. Only one significant interaction (workplace bullying x changing view) was found, which partly confirmed the hypothesis. The findings in this study also indicate resilience, seeking help, and changing views have preventive factors against subsequent psychological distress even after controlling psychological distress at baseline. On the other hand, negative coping styles such as avoidance increased psychological distress. These results partly support the hypotheses that individual and organizational resources protect bullied employees against developing psychological distress. However, psychological distress at baseline had the most substantial effect on future psychological distress independently from workplace bullying. This indicates current distress would last or even worsen employees’ mental health if there is no intervention.

A significant moderating effect of changing mood on the association between bullying and psychological distress was found in this study. As hypothesized, this indicates that emotional coping style could deteriorate mental health when they are bullied. Changing mood is one of the emotion-focused coping [38]. Emotion-focused coping strategies have been reported to be suitable when the stressors are unchangeable [28]. The study results are in line with the study for cyberbullied children that showed emotion-focused cyber-specific coping was associated with more health complaints and depressive feelings [39]. Although there are not so many studies investigating the moderating effect of coping styles on the association between workplace bullying and mental health [32], this study adds a piece of evidence that negative coping styles such as emotional coping may worsen mental health outcomes due to workplace bullying. The reason why emotion-focused coping style did not prevent adverse health effects may be because this coping style does not solve the relationships between victims and perpetrators but rather avoid from the situation [39]. Although significant interaction effects were not found in this study, the results showed that positive coping styles such as seeking solutions could prevent subsequent psychological distress, independently of exposure to bullying. Further longitudinal studies are needed to clarify the moderating effect of coping styles on bullying and mental health outcomes.

Personal resilience had a main effect on reducing subsequent psychological distress even though participants experienced workplace bullying. The results agree with cross-sectional studies reporting that employees with higher levels of resilience have lower levels of emotional exhaustion, distress, or physical strain when exposed to bullying at work [23–26]. On the other hand, a significant interaction of workplace bullying and resilience on psychological distress was not found in this study, which rejected H2. This means even those who had high resilience had higher subsequent psychological distress when they were bullied, compared to those who were not bullied. In other words, workplace bullying itself or baseline psychological distress is a strong predictor of adverse mental health even among high resilient employees, which coincides with various longitudinal studies reporting a strong association between workplace bullying and mental health outcomes [9, 40]. The results of this study also showed that resilience was negatively associated with workplace bullying. This is probably because experience of workplace bullying may affect personal resilience because workers who were bullied tend to feel helpless and lose their confidence [41].

Seeking help for a solution and changing a point of view were significantly negatively associated with subsequent psychological distress when participants experienced workplace bullying, while avoidance was significantly positively associated. In contrast, active solution and changing mood were not significantly associated with psychological distress. The results are compatible with the cross-sectional study that reported seeking help moderated the relationship between bullying and psychological well-being, while avoidance and doing nothing have negative impacts on the relationship [31]. The possible reason why active solution did not have a protective factor against psychological distress is that it is usually difficult for victims to change perpetrators or working environments directly due to the power imbalance nature of bullying [42]. This indicates that active coping styles are effective for general “resolvable” work stressors but not for workplace bullying, which usually cannot be solved easily by victims as mentioned earlier.

Among various social support, only co-worker support reduced psychological distress under exposure to bullying, only before controlling psychological distress at baseline. This is consistent with the study that only coworker support moderates the relationship between workplace bullying and mental health [14]. However, an interaction effect of coworker support and bullying on psychological distress was not observed in this longitudinal study, which rejected H1. Moderating effects of social support on the association between workplace bullying and mental health is also inconclusive in the literature [13, 15, 16]. This might be due to the nature of social support. As indicated in matching hypothesis, social support buffers distress only when the support matches the recipient’s needs [43]. Receiving support does not mean the victim gets enough support they want.

Several limitations in this study should be taken into consideration when interpreting the results. First, this study did not assess the moderating effects of other variables, such as psychological capital, job demands, or job control, on the association between workplace bullying and psychological distress. Second, factors that could influence workers’ perceptions of bullying, such as neuroticism was not controlled. Third, although common method bias was minimized in the longitudinal design by measuring exposure and outcome variables at different periods, some amount of common method bias might still occur in a questionnaire survey since all measures were self-reported. Lastly, the response rate at baseline was 83.9% and the follow-up rate was 77.2%, which means a total of 35% of the employees did not participate in the follow-up survey. Since statistical differences were found between follow-ups and drop-outs, there may be a selection bias in this study.

The strength of our study is revealing a longitudinal moderating effect of individual and occupational resources on the association between workplace bullying and psychological distress using a relatively large sample with diverse professions. Past researchers have investigated these moderating effects only in a cross-sectional design, which increase the possibility of common method bias. This also leads to an overestimation of the association of moderating effects of individual and occupational resources on the association between workplace bullying and psychological distress. This study made it possible to overcome these methodological issues using a longitudinal design. The findings of this study offer a new perspective on work-related bullying and mental health research.

## Conclusions

The present findings suggest that emotional coping style, i.e., changing mood, boosts their future psychological distress when bullied. This means changing mood is not appropriate or even a harmful coping style or advice for victims of workplace bullying. Furthermore, one should take note that no buffering effect of positive coping styles, resilience, worksite social support, and family/friends support was found on bullying and psychological distress. This indicates preventing workplace bullying should be prioritized in organizations rather than building the resilience of victims or enhancing social support for victims. Also, the strongest predictor of psychological distress at one-year follow-up was baseline psychological distress, indicating early intervention to reduce psychological distress is beneficial for employees’ future mental health. Human resource personnel and occupational health professionals should monitor employees’ mental health status and intervene to prevent prolonged persistent distress.

## Data Availability

The datasets used and/or analyzed during the current study are available from the corresponding author on reasonable request.

## Abbreviations

BJSQ: Brief Job Stress Questionnaire
BSCP: Brief Scales for Coping Profile
CD-RISC: Connor–Davidson Resilience Scale
NAQ-R: Negative Acts Questionnaire-Revised
